# Supplementary material for: Rhodelphis edaphicus sp. nov.—a new lineage of predatory archaeplastids from agricultural soil
Source: PeerJ. 2025 Oct 1;13:e20071. doi: 10.7717/peerj.20071 (PMC12495952; doi:10.7717/peerj.20071)
Supplement: Supplemental Information 1 [file peerj-13-20071-s001.docx]

## Table 1. Similarity of 18S rRNA gene sequences among *Rhodelphis* species (%)

|  | *R. mylnikovi* | *R. limneticus* | *R. marinus* | *R. edaphicus* sp. nov. |
| --- | --- | --- | --- | --- |
| *R. mylnikovi* | 100 | 95.16 | 91.11 | 92.78 |
| *R. limneticus* |  | 100 | 91.18 | 92.7 |
| *R. marinus* |  |  | 100 | 89.86 |
| *R. edaphicus* sp. nov. |  |  |  | 100 |
